# Myocardial biomechanical effects of fetal aortic valvuloplasty

**DOI:** 10.1007/s10237-024-01848-0

**Published:** 2024-04-29

**Authors:** Laura Green, Wei Xuan Chan, Andreas Tulzer, Gerald Tulzer, Choon Hwai Yap

**Affiliations:** 1 Department of Biomedical Engineering, Imperial College London, L2 Bessemer Building, South Kensington Campus, London, SW7 2AZ UK; 2 BHF Centre of Research Excellence, Imperial College London, London, UK; 3 Department of Pediatric Cardiology, Children’s Heart Center Linz, Kepler University Hospital, Linz, Austria; 4 Medical Faculty, Johannes Kepler University Linz, Altenberger Strasse 69, 4040 Linz, Austria

**Keywords:** Fetal aortic valvuloplasty, Fetal aortic stenosis with evolving hypoplastic left heart syndrome, Patient-specific finite element modeling, Virtual intervention analysis

## Abstract

Fetal critical aortic stenosis with evolving hypoplastic left heart syndrome (CAS-eHLHS) can progress to a univentricular (UV) birth malformation. Catheter-based fetal aortic valvuloplasty (FAV) can resolve stenosis and reduce the likelihood of malformation progression. However, we have limited understanding of the biomechanical impact of FAV and subsequent LV responses. Therefore, we performed image-based finite element (FE) modeling of 4 CAS-eHLHS fetal hearts, by performing iterative simulations to match image-based characteristics and then back-computing physiological parameters. We used pre-FAV simulations to conduct virtual FAV (vFAV) and compared pre-FAV and post-FAV simulations. vFAV simulations generally enabled partial restoration of several physiological features toward healthy levels, including increased stroke volume and myocardial strains, reduced aortic valve (AV) and mitral valve regurgitation (MVr) velocities, reduced LV and LA pressures, and reduced peak myofiber stress. FAV often leads to aortic valve regurgitation (AVr). Our simulations showed that AVr could compromise LV and LA depressurization but it could also significantly increase stroke volume and myocardial deformational stimuli. Post-FAV scans and simulations showed FAV enabled only partial reduction of the AV dissipative coefficient. Furthermore, LV contractility and peripheral vascular resistance could change in response to FAV, preventing decreases in AV velocity and LV pressure, compared with what would be anticipated from stenosis relief. This suggested that case-specific post-FAV modeling is required to fully capture cardiac functionality. Overall, image-based FE modeling could provide mechanistic details of the effects of FAV, but computational prediction of acute outcomes was difficult due to a patient-dependent physiological response to FAV.

**Supplementary Information:**

The online version contains supplementary material available at 10.1007/s10237-024-01848-0.

## Introduction

Fetal hearts diagnosed with critical aortic stenosis with evolving hypoplastic left heart syndrome (CAS-eHLHS) present physiological, functional and morphological aberrations, such as elevated left ventricle (LV) pressure, reduced LV stroke volume, retrograde transverse aortic arch flow, reduced myocardial strains, globular LV structures, endocardial fibroelastosis, monophasic mitral valve (MV) inflow and the presence of mitral valve regurgitation (MVr) (Freud et al. 2014; Friedman et al. 2014; Ishii et al. 2014; Mäkikallio et al. 2006; Tulzer et al. 2022). Such aberrations can lead to adverse remodeling in gestation, with a natural history study showing there to be a 73% likelihood of progression from CAS-eHLHS to hypoplastic left heart syndrome (HLHS) by the time of birth (Gardiner et al. 2016; Mäkikallio et al. 2006).

Fetal aortic valvuloplasty (FAV) is a mid-gestation in utero catheter-based procedure, undertaken on CAS-eHLHS patients, to widen the stenotic aortic valve (AV). Under ultrasound guidance the balloon catheter is inflated across the stenotic AV multiple times, to widen the valve, allowing increased blood flow, to promote healthier development in gestation. Patient selection criteria for FAV varies among institutes; however, commonly, if the fetus meets the criteria for evolving hypoplastic left heart syndrome (eHLHS), which is moderate to severe LV dysfunction, retrograde aortic arch flow and the presence of left-to-right foramen ovale shunting (Friedman et al. 2018), with the inclusion of functional metrics such as an LV long axis Z score > − 1.0 (Tulzer et al. 2022), the fetus will be selected for FAV. Through retrospective studies FAV has been shown to promote healthier development in gestation, by reducing the likelihood of a HLHS outcome (Friedman et al. 2018; Gardiner et al. 2016; Mäkikallio et al. 2006; Tulzer et al. 2022).

Following a technically successful FAV, LV myocardial strains were shown to increase (Ishii et al. 2014) and LV function was shown to significantly improve (Wohlmuth et al. 2016). The functional improvements, post-FAV, highlight the LVs ability to biomechanically respond to the essentially mechanical procedure of FAV. However, there is still limited in-depth understanding of the biomechanical effects of FAV on the LV myocardium. For example, it is not completely clear how much stenosis relief is generally achieved, how various extents of stenosis relief affect LV pressure, stroke volume and myocardial strains, and how much reduction of abnormally high LV and LA pressures can be achieved. Furthermore, a side-effect of FAV is aortic valve regurgitation (AVr) (Arzt et al. 2011), as FAV tended to damage the AV, but the effects of AVr on myocardial biomechanics and function are unclear.

We previously studied the fluid mechanics of CAS-eHLHS disease (Wong et al. 2022) and FAV intervention (Wong et al. 2023). Our findings revealed aberrant flow patterns and energy dynamics occur during disease (Wong et al. 2022) and demonstrated that FAV intervention could enhance flow rates but led to inefficient energy dynamics (Wong et al. 2023). However, these studies could not elucidate myocardial tissue biomechanics, which must be studied to fully understand the biomechanical implications of disease and intervention.

Image-based finite element (FE) computational modeling is a robust approach for simulating the contractile ability, pumping function, and biomechanics details of the myocardium (Shavik et al. 2018; Zheng et al. 2022, 2023), helping comprehend variations in stresses and strains, and compute the stroke volume and pressure of the heart during remodeling due to disease or interventions. FE modeling of the fetal heart has previously aided better understanding of the biomechanics of the fetal heart as well, providing an understanding of biomechanical disease features during CAS-eHLHS (Green et al. 2022, 2024; Ong et al. 2020), where we determined that biomechanics parameters such as myocardial stress can be good predictors of FAV intervention outcomes (Green et al. 2024).

However, detailed FE simulation of FAV intervention has not been performed previously, and we have limited understanding of the mechanistic effects of the intervention on fetal LV function and biomechanics and how the LV myocardium would respond to intervention. Therefore, here, we performed image-based, patient-specific FE modeling of CAS-eHLHS fetal hearts to address this. We first performed virtual FAV (vFAV) based on pre-FAV scans to determine the effects of stenosis relief alone. We then compared the results from FE modeling of patient-specific pre- and post-FAV models to investigate specific physiological changes across FAV.

## Methods

### Image acquisition

4D echocardiography images of 4 CAS-eHLHS fetal hearts pre- and post-FAV were acquired using the GE Voluson E10 (GE Healthcare, Chicago, IL, USA) ultrasound machine. Images were taken in the spatiotemporal image correlation mode, at sweeps of 10–15 s and a capture rate of 70–90 frames per second. All images were obtained with informed consent from the Kepler University Hospital, Austria, under Institutional Review Board protocol 1009/2017.

### Patient characteristics

We selected the first 4 patients (2 with a univentricular (UV) birth outcome and 2 with a biventricular (BV) outcome) that became available, where clinical scans and measurements were complete and did not impose any specific selection criteria. The characteristics of the patients included in the study are described in Table 1. vFAV simulation methods used the age at FAV; patient-specific post-FAV simulation methods used the age at the post-FAV scans.

**Table 1:** Patient characteristics of CAS-eHLHS cases before the procedure

	Patient 1	Patient 2	Patient 3	Patient 4
Age at FAV (wks + days)	24 + 6	29 + 3	22 + 4	24 + 6
Age at Post-FAV scan (wks + days)	25 + 0	30 + 2	22 + 6	25 + 0
Postnatal circulation	BV	BV	UV	UV
Multiple FAVs	N	N	N	N
Bradycardia	Y	N	N	Y
Pericardial effusion	N	N	N	N
LV thrombus	N	N	Y	N
Hydrops	N	N	N	N
Postnatal procedures	Dil	RK	NW	NW

*Y* yes, *N* no, *Dil* balloon dilation of the AV, *RK* Ross-Kono procedure, *NW* Norwood procedure

### Image processing

**Fig. 1:**
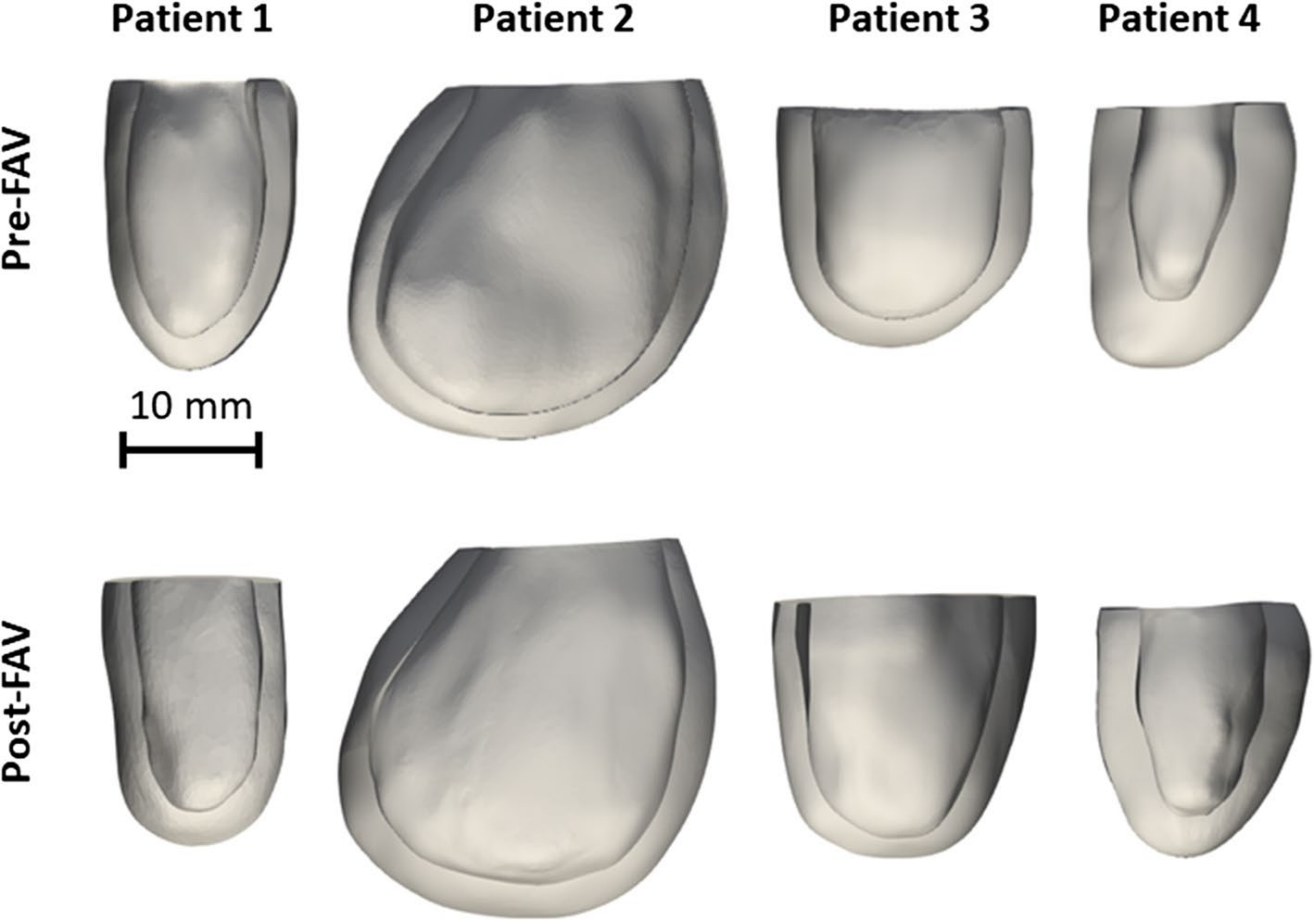
Patient-specific geometries, reconstructed from pre- and post-FAV echocardiographic data

For patient-specific FE simulation of both pre-FAV and post-FAV LVs, 3D reconstructions of the LV myocardium, left atrium (LA) cavity and right ventricle (RV) cavity were first performed as previously reported (Green et al. 2022). Briefly, 2D slices were extracted from the 4D volume files, and then, binary segmentation of the myocardium was performed using a lazy snap algorithm (Li et al. 2004). 3D reconstruction was then performed with VMTK (www.vmtk.org), with the reconstructed geometries smoothed in Geomagic (Geomagic Inc., Morrisville, NC, USA). 3D motions of the LV, LA and RV were extracted using a validated cardiac motion estimation algorithm (Wiputra et al. 2020). The algorithm modeled motion in the image with spatial b-splines of temporal Fouriers, and was curve-fitted to the displacement fields from 3D pair-wise registration images of consecutive time points. The final reconstructed LV geometries are depicted in Fig. 1, where satisfactory tracking can be demonstrated in Supplementary Fig. S1.

LV myocardial Green strains (Eq. 1) were computed from the extracted cardiac motions, in both the longitudinal and circumferential directions, across the entire heart, at the mid-wall location (in between the endocardial and epicardial surfaces), before being spatially averaged. The mid-wall location was identified by averaging the epicardial and endocardial boundaries (example in Supplementary Material Section 1.0).

1 $${\text{Green lagrangian strain = }} \frac{1}{2}\left( {F^{T} \cdot F - I} \right).$$

where $F$ was the deformation gradient tensor and $I$ the identity matrix.

### FE and lumped parameter modeling formulation

Image-based, patient-specific FE modeling of fetal LV myocardial biomechanics was performed in accordance with our previous methodologies (Green et al. 2022; Ong et al. 2020), where the FE model was connected to a lumped parameter model to enable ventricular-vascular coupling (Pennati et al. 1997; Pennati and Fumero 2000). The lumped parameter model used was based on Pennati et al.’s work (Pennati et al. 1997; Pennati and Fumero 2000) and was age scalable to a range of gestational ages, through a series of allometric equations. The model underwent minor recalibration using descending aorta pulse pressure measurements (Versmold et al. 1981) and more recent measurements of human fetal intracardiac pressure (Johnson et al. 2000), further details have been supplied in Supplementary Material Section 3.0. Our FE and lumped parameter codes have been made available at https://github.com/WeiXuanChan/heartFEM.

The behavior of the myocardium was described via a transversely isotropic Fung type passive stiffness model (Guccione et al. 1991) and an active tension model (Guccione et al. 1993). The Fung type transversely isotropic passive stiffness model (Guccione et al. 1991) was described via the following strain energy function, $W$:

2 $$W = \frac{1}{2}C\left( {e^{{b_{ff} E_{ff}^{2} + b_{xx} \left( {E_{ss}^{2} + E_{nn}^{2} + E_{sn}^{2} + E_{ns}^{2} } \right) + b_{fx} \left( {E_{fn}^{2} + E_{nf}^{2} + E_{fs}^{2} + E_{sf}^{2} } \right)}} - 1} \right),$$

where $C$ was the passive stiffness coefficient, $E$ was the Green–Lagrange Strain, with subscripts $f$, $s$ and $n$ denoting the fiber, sheet and sheet-normal directions, respectively, and $b$ was the stiffness exponent in specific directions specified by its subscript. The values assigned to stiffness parameters are shown in Table 2 and were obtained from a previous fetal heart FE study (Ong et al. 2020). Although myocardial stiffness could typically be scaled up or down via the stiffness coefficient ($C$ in Eq. 2) to model person-to-person variability in myocardial stiffness, Ong et al. showed that large changes to $C$ had minimal effect on peak systolic pressure, ventricular size, stroke volume and peak systolic myofiber stress (Ong et al. 2020). As such, we used the same stiffness model in Table 2 for all simulation scenarios in this study.

**Table 2:** Finite element modeling parameters required for both the passive and active description of the myocardium (Guccione et al. 1991, 1993)

Parameter	Symbol	Unit	Value
Passive stiffness coefficient	C	Pa	200
Stiffness coefficient in fiber direction	${b}_{ff}$	–	29.9
Stiffness coefficient in sheet and sheet normal direction	${b}_{xx}$	–	13.3
Stiffness coefficient in shear directions	${b}_{fx}$	–	26.6
Shape of peak isometric tension-sarcomere length relation	*B*	μm^−1^	4.75
Sarcomere length at which active tension becomes zero	${l}_{0}$	μm	1.58
Relaxed sarcomere length	${l}_{r}$	μm	1.85
Time intercept of linear relaxation duration with sarcomere length	*b*	ms	-800
Gradient of linear relaxation duration with sarcomere length relaxation	*m*	ms μm^−1^	524
Maximum intracellular calcium concentration	${Ca}_{0}$	μM	4.35

The Guccione’s active tension model, ${P}_{{\text{act}}}$ (Guccione et al. 1993), was expressed as:

3 $$P_{{{\text{act}}}} = T_{{\text{0,LV}}} \frac{{{\text{Ca}}_{0}^{2} }}{{{\text{Ca}}_{0}^{2} + {\text{ECa}}_{50}^{2} }}C_{t} ,$$

where ${T}_{0,{\text{LV}}}$ described the maximum fiber tension, which described myocardial contractility and could be back computed in the patient-specific optimization methods. Furthermore, the maximum intracellular calcium concentration, ${{\text{Ca}}}_{0}$, was the value of sarcomere length-dependent calcium sensitivity, and ${{\text{ECa}}}_{50}$ was the length-dependent calcium sensitivity variable, which was calculated as follows:

4 $${\text{ECa}}_{50} = \frac{{{\text{Ca}}_{0} }}{{\sqrt {\exp \left[ {B\left( {l - l_{0} } \right)} \right] - 1} }},$$

where $B$ described the shape of peak isometric tension-sarcomere length relation, $l$ was the sarcomere length (calculated via strain outputs of the FE), and ${l}_{0}$ was the sarcomere length at which there was no longer any tension generation, all of which are documented in Table 2. ${C}_{t}$ in Eq. 3 was the temporal variation of the calcium activation model, which was described as:

5 $$C_{t} = \frac{1}{2}\left( {1 - \cos \omega } \right),$$

where $\omega$ was dependent on the cycle time, with the variation:

6 $$\omega = \left\{ {\begin{array}{*{20}c} {\pi \frac{t}{{t_{0} }}\;{\text{when }}\;0 \le t < t_{0} ,} \\ {\pi \frac{{t - t_{0} + t_{r} }}{{t_{r} }}\;{\text{when}}\;t_{0} \le t < t_{0} + t_{r,} } \\ {0\;{\text{when}}\;t_{0} + t_{r} \le t,} \\ \end{array} } \right.$$

where ${t}_{0}$ was the time to peak tension, calculated using the model-specific cardiac cycle length and Mulieri et al.’s measured relationship between cardiac cycle and time to peak tension (Mulieri et al. 1992). Furthermore, ${t}_{r},$ was the relaxation time and was calculated using the following equation:

7 $$t_{r} = ml + b,$$

where $m$ was the gradient of linear relaxation duration and b was the time intercept of linear relaxation duration, dependent on the degree of myocyte stretch, with parameter values also documented in Table 2.

The myocardium helix angle configuration was assumed to be the same for all patients and was assigned based on the average of 3 previous publications, which quantified the fetal helix angle configuration (Garcia-Canadilla et al. 2018; Nishitani et al. 2020; Ohayon et al. 1999). Therefore, the myocardium helix angle configuration was assumed to vary linearly from − 52° at the epicardium to + 71° at the endocardium.

To calculate the unloaded (zero chamber pressure) state of the LV, we adopted Finsberg et al.’s backward displacement method (Finsberg et al. 2018). This involved iteratively simulating the unloading of an initial diastolic LV geometry to the load-free state (estimated as the inverse deformation of loading of a similar pressure) followed by the diastolic pressure loading of the load-free state to end-diastolic state. When the iterative simulations were conducted, the pressure of the initial LV geometry was adjusted, and iterations were repeated until the end-diastolic state achieved the targeted pressure and volume. In CAS-eHLHS cases, the LA was often pressurized and enlarged (Tulzer et al. 2022), but no quantification of LA pressure is available. Therefore, we performed a simplistic patient-specific estimation of end-diastolic LA pressure, by assuming a linear relationship between LA size and pressure (Matsuda et al. 1990; Wong et al. 2022), which was described as:

8 $${\text{LV end diastolic pressure = }}\frac{{\text{Minimum LA volume}}}{{\text{LA capacitance}}}{.}$$

where LA capacitance was assumed based on the gestational age-specific value derived in the lumped parameter model (Pennati et al. 1997; Pennati and Fumero 2000). LV myocardium models, displayed in Fig. 1, were meshed with a minimum of 2500 quadratic tetrahedral elements, which was sufficient for mesh convergence as shown in our previous study (Ong et al. 2020). The formal FE simulation was performed by minimizing the weak formulation of a Lagrangian function described by Shavik et al., which enforced tissue stress equilibrium, incompressibility, and required a specific cavity volume to yield cavity pressure, using the Newton Solver in the FEniCS software (Shavik et al. 2018). The boundary conditions were like that previously reported (Shavik et al. 2018), with the basal plane of the LV constrained in the longitudinal direction and a weak 90 Pa spring applied to the entire epicardium at the load-free state, to constrain translational motion of the model and to imitate the behavior of the surrounding tissues. The model was executed for 30 cycles, to ensure steady state was achieved.

### vFAV methods

vFAV was performed by modulating the lumped parameter model of the patient-specific image-based FE models of pre-FAV fetal LVs previously established (Green et al. 2024). To conduct patient-specific FE modeling of pre-FAV LVs, the lumped parameter model was first scaled to the specific pre-FAV gestational age. Iterative FE simulations were then conducted, varying dissipative coefficients of AV outflow, MV inflow and MVr flow, as well as peak myocardial active tension, until the simulated valvular pressure gradients ($\Delta P$) matched echo-measured gradients, which were calculated using the simplified Bernoulli equation and Doppler velocities, and the simulated stroke volume matched that from echo images. Through this matching process, the patient-specificity of the model was maintained, and the valve dissipative coefficients and peak myocardial active tension could be back-computed. Valve dissipative coefficients had to be back-computed instead of being computed from $\Delta P$ and flow rate ($Q$), because $Q$ could not be determined from echo, as valve velocity measurements could not be converted to $Q$ because valve orifice area was not measurable from echo, and LV stroke volume could not be used to infer inflow or outflow rates due to regurgitation and thus multiple in/outlets. Therefore, in Eq. 9, $K$, the valve dissipative coefficient, described the resistance of the heart valves to flow, like so:

9 $$\Delta P = K \cdot Q^{2} .$$

This model was adopted from (Pennati et al. 1997). Peak myocardial active tension was initially set as 60 kPa based on the upper limit of experimental measurements (Racca et al. 2016).

**Fig. 2:**
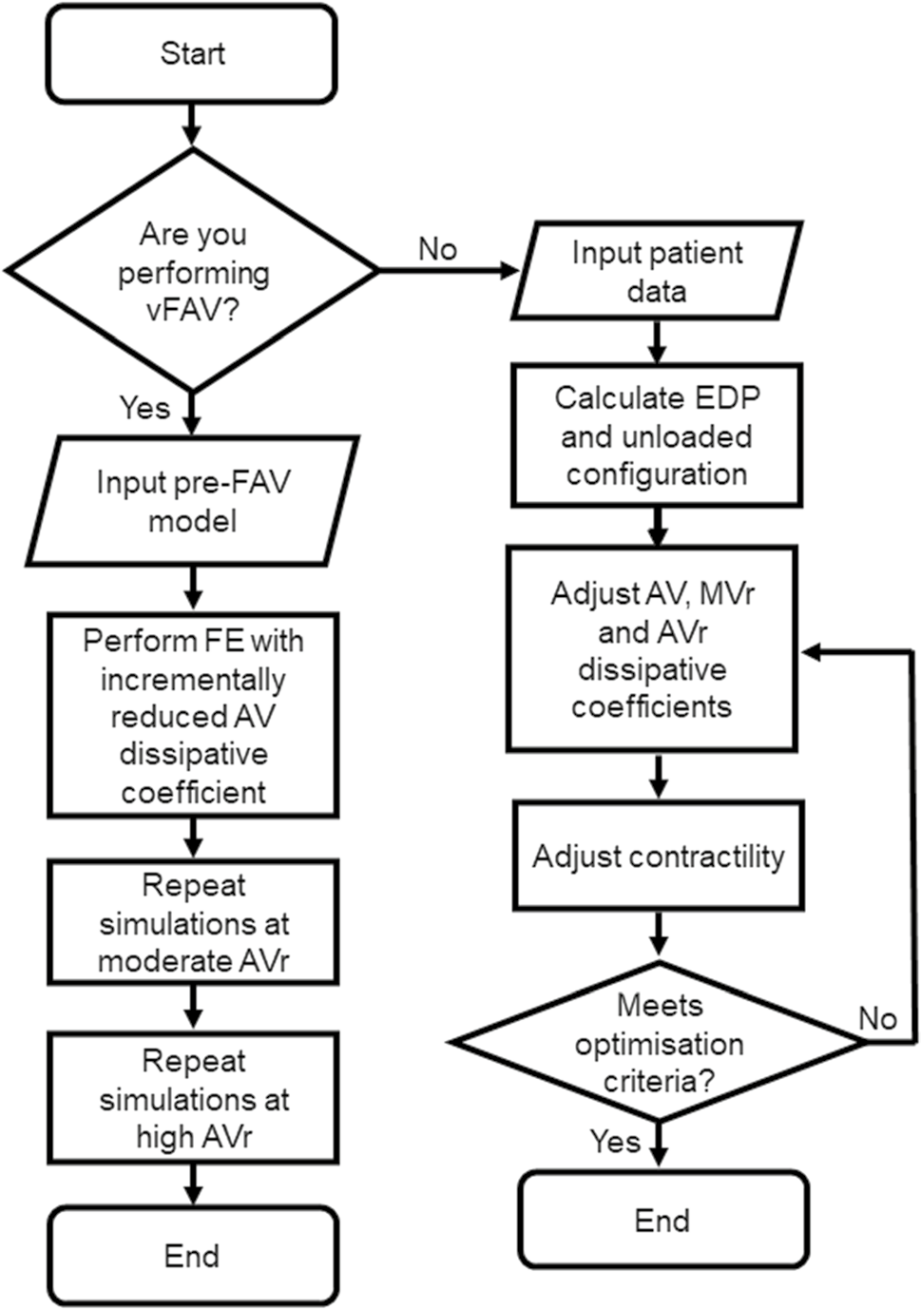
Description of modeling protocol for vFAV intervention and patient-specific post-FAV intervention modeling steps

During the patient-specific matching optimizations, a gradient descent algorithm was used to optimize the Doppler gradients for MV, MVr and AV, to simulated pressure gradients, while another gradient descent algorithm was used to optimize the match between simulated and imaged stroke volume (Fig. 2). The solution was deemed convergent if errors for all parameters were < 10%. If this was not achieved, the age scaling of the lumped parameter model was adjusted, and the iterative matching process repeated. This age scaling adjustment represented a way to adjust for fetal body size variability or body developmental maturity.

From the optimized pre-FAV models, AV dissipative coefficient was reduced to simulate the widening of the AV and relief of stenosis via FAV. A few magnitudes of reduction were simulated, between the valvular dissipative coefficient value of the pre-FAV LV to that of an age-matched healthy LV, as defined in our lumped parameter model (Wong et al. 2022). The process of simulating relief of the AV was then repeated for two levels of AVr severity, because a range of AVr velocities were possible, as indicated by the relatively wide standard deviation of regurgitation velocity (1.92 ± 0.87 m/s) from our clinical database of 44 cases (Supplementary Material Fig. S4). The two levels of AVr severity were computationally assigned as such: moderate AVr, where the regurgitant valve dissipative coefficient was equal to the AV antegrade flow, and high AVr, where the regurgitant valve dissipative coefficient was 10× that of the AV antegrade flow. By this definition, a more severe AVr had a higher regurgitation flow valvular dissipative coefficient that would translate to higher AVr velocity via Bernoulli’s equation. This fitted with the clinical approach, where severity of AVr was defined by regurgitation velocity magnitude. An overview for the vFAV modeling protocol is described in Fig. 2.

A sensitivity analysis was performed to assess how other physiological features (such as a change in heart rate, increased vascular resistance, and increased myocardial contractility) affected valve velocities and stroke volume, and how they contributed to the match between vFAV and image-based measurements.

### Patient-specific post-FAV methods

The patient-specific post-FAV FE modeling was conducted under the same protocol as that for pre-FAV FE modeling (Green et al. 2024), with the addition of matching the patient-specific AVr ΔP, as post-FAV hearts have AVr but pre-FAV hearts did not, an overview of the modeling protocol is included in Fig. 2.

### Computational biomechanics characteristics

From the FE and lumped parameter modeling methods a series of biomechanics parameters could be output to aid analysis of the models generated. Stroke volume was extracted based on the cardiac motion estimation algorithm and peak LV and LA pressure were extracted directly from the computational methods. Peak myofiber stress was calculated by computing the stress in the myofiber direction (direction of the helix angle) at the time point of peak LV systolic pressure and then was spatially averaged over the entire myocardial volume, as follows,

10 $${\text{Peak myofiber stress}} = \frac{1}{V}\mathop \int \limits_{V} \overrightarrow {{f_{d} }} \sigma \overrightarrow {{f_{d} }} dV,$$

where *V* was the volume of the myocardium, $\overrightarrow {{f_{d} }}$ was the unit vector in the myocardial direction, and $\sigma$ was the stress tensor at peak systole. Work done was quantified by calculating the area inside the pressure volume (PV) loop and described the ability of the LV to eject fluid. Valvular velocity was calculated by extracting $\Delta P$ across each valve, from the lumped parameter model, and then, velocity was computed via the simplified Bernoulli equation.

## Results

### Effects of FAV on fetal heart biomechanics and function

Supplementary Table S2 showed that a good match was obtained for the pre-FAV models that were optimized for patient-specific characteristics. From the optimized pre-FAV models, vFAV was simulated by reducing the valvular dissipative coefficient of the stenotic AV, and by introducing AVr, which commonly occurred after the procedure (Bradford et al. 2022). The vFAV altered only AV flow dissipative coefficients and introduced AVr at 2 magnitudes, every other parameter was kept at the pre-FAV level, so that we isolated and understood the biomechanical effects specific to this modulation of the AV. In our previous study, we found that the reduction in stenosis via FAV could be variable and span a range of magnitudes (Wong et al. 2023). As such, we conducted simulations over a range of reduced AV flow dissipative coefficients.

**Fig. 3:**
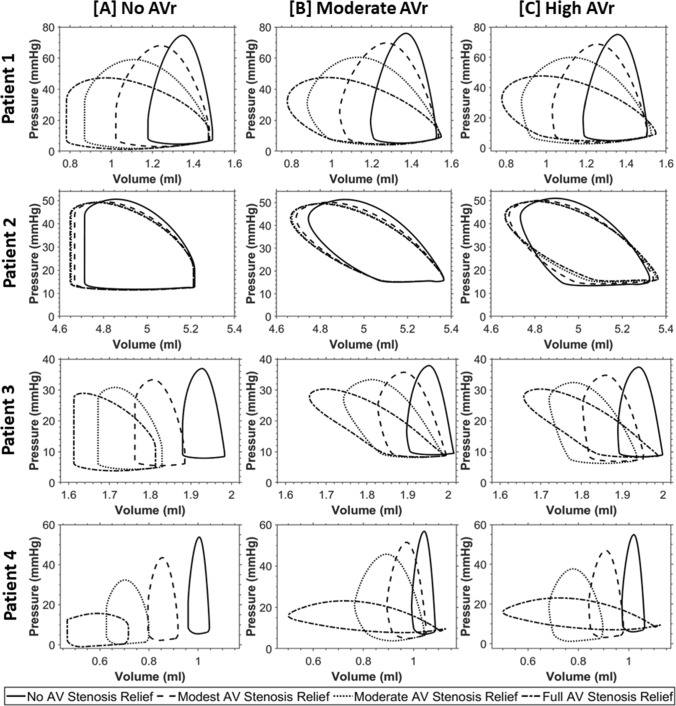
PV loops from patient-specific pre-FAV simulations of 4 patients, along with those from simulations of vFAV. On top of the baseline disease scenario, 3 levels of stenosis relief were simulated for each FAV case, modest, moderate, and full relief of stenosis. At the same time, 3 levels of AVr after FAV were simulated, [**A**] no AVr, [**B**] moderate AVr, and [**C**] high AVr

PV loop results from vFAV simulations are shown in Fig. 3. Generally, we observed that relieving the AV stenosis led to reduced LV pressure and greater stroke volume, the extent of which directly depended on the level of stenosis relief. Different patients also experienced different extents of LV depressurization and stroke volume augmentation, which were likely dependent on the stenosis severity. Patient 2, for example, had a milder stenosis, based on the log ratio of pre-FAV to healthy AV dissipative coefficient, documented in Table 3, and thus did not undergo a drastic change after FAV. When AVr was introduced (Fig. 3B, C), the LV effectively had regurgitation in both the MV and AV, and the PV loops no longer exhibited isovolumic periods and appeared slanted. AVr caused the LV to fill more during diastole, leading to an increase in end-diastolic volume, thus increasing stroke volume. This increase could be drastic, like that observed in Patients 3 and 4 in Fig. 3. AVr also increased end-diastolic pressure, due to the greater filling volume, and slightly increased peak systolic pressure, likely due to additional engagement of contractile myofibers, resulting from the greater filling volume. This meant that the atria pressure could fail to decrease from the diseased baseline and, in some instances, increase further.

**Table 3:** Valve dissipative coefficient in patient-specific, image-based pre- and post-FAV simulations that enabled the best match between imaged and simulated stroke volumes and valve velocities

ID	Scenario	AV dissipative coefficient (mmHg s^2^/ml^2^)	MV dissipative coefficient (mmHg s^2^/ml^2^)	Disease severity metric (log ratio of pre-FAV to healthy AV dissipative coefficients)
Patient 1	Pre-FAV	13.69	0.1168	3.35
	Post-FAV	3.513	0.1210	2.76
	Age-matched Healthy LV	0.006174	0.01235	
	vFAV	0.006174 to 13.69	0.1168	
Patient 2	Pre-FAV	0.2135	0.02420	1.90
	Post-FAV	0.2052	0.03817	1.88
	Age-matched Healthy LV	0.002705	0.005411	
	vFAV	0.002705 to 0.2135	0.02420	
Patient 3	Pre-FAV	33.07	0.2960	3.52
	Post-FAV	0.2618	0.1247	1.41
	Age-matched Healthy LV	0.01008	0.02015	
	vFAV	0.01008 to 33.07	0.2960	
Patient 4	Pre-FAV	151.2	2.9235	4.39
	Post-FAV	1.6222	0.2030	2.42
	Age-matched Healthy LV	0.006174	0.01235	
	vFAV	0.006174 to 151.2	2.9235	

Valve coefficient from an age-matched healthy fetal heart, determined via our scalable fetal circulatory lumped parameter model for healthy fetuses (Wong et al. 2022), was included for comparison, as well as valve coefficients used in our vFAV simulations, which ranged from the best fit pre-FAV diseased value to that of healthy LV value. The log ratio of the pre-FAV to healthy AV dissipative coefficient was quantified to estimate disease severity, where Patient 2 exhibits the lowest ratio and therefore had the lowest disease severity in the cohort studied

**Fig. 4:**
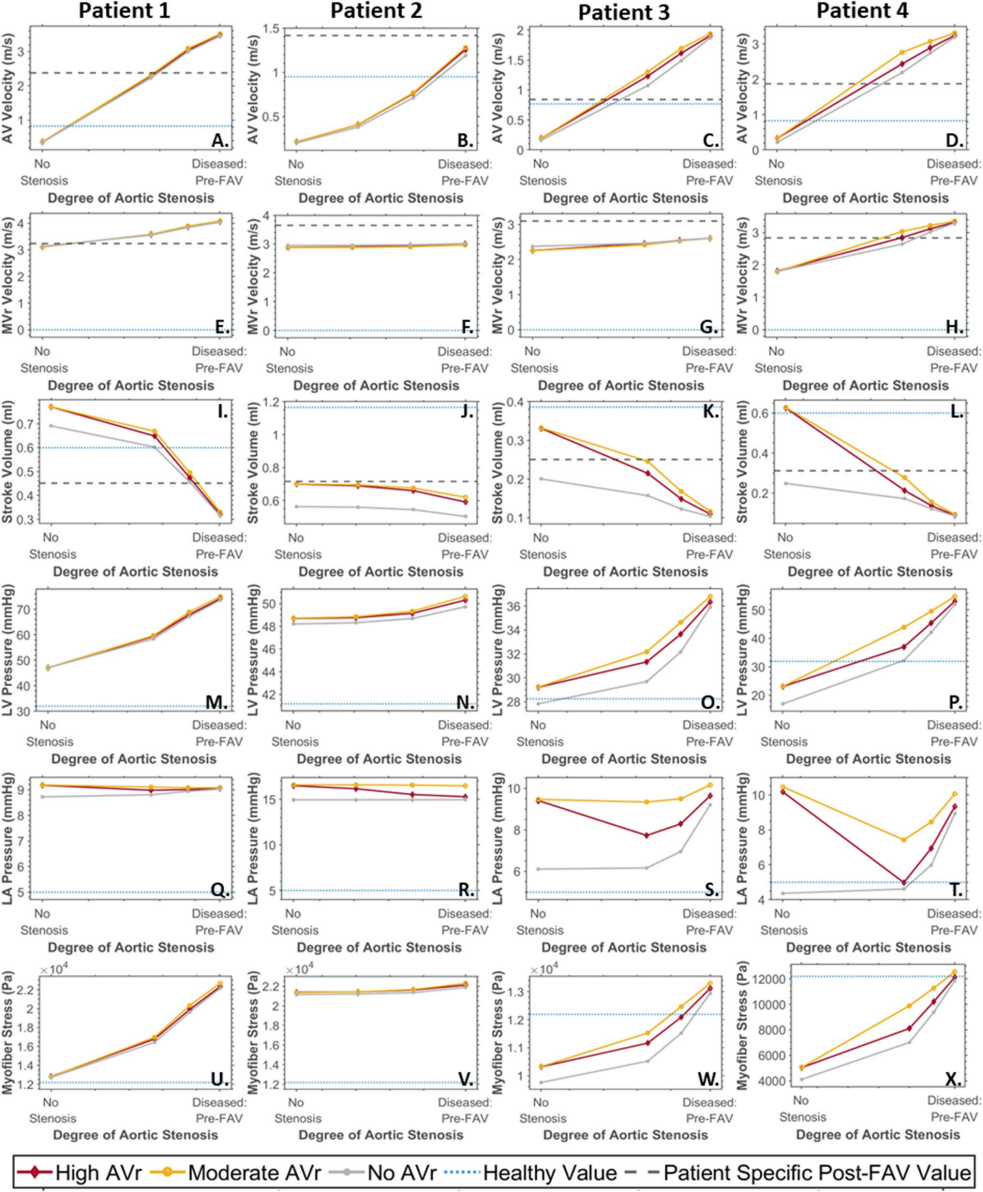
Cardiac physiological characteristics after vFAV, from image-based FE simulations, including **A**–**D** AV velocity, **E**–**H** MVr velocity, **I**–**L** stroke volume, **M**–**P** peak systolic LV pressure, **Q**–**T** end diastolic LA pressure and **U**–**X** peak myofiber stress. Characteristics were plotted across a range of aortic stenosis dissipative coefficients, which corresponded to different extents of stenosis relief from FAV, where the right end of the horizontal axis was the baseline diseased condition, while the left end of the axis was the no stenosis scenario, where the AV dissipative coefficient was that of an age-matched healthy subject. 3 levels of AVr were simulated: no AVr (gray line), moderate AVr (yellow line), and high AVr (red line). Values for an age-matched healthy subject were included in all plots (blue dotted line), while the AV and MVr velocity and stroke volume extracted from the patient-specific post-FAV values were plotted in a gray dashed line (**A**–**L**)

Further analysis of vFAV biomechanics outputs was conducted and compared with the parameter values for the healthy fetal hearts, which have been indicated by the horizontal blue dotted line in Fig. 4. These age-dependent healthy parameters were obtained from Parasuraman et al. for AV velocity measurements ranging from 12 to 40 weeks gestation (n = 221) (Parasuraman et al. 2013), from Johnson et al. for intracardiac pressure measurements during peak systole and end diastole, across 16–29 weeks gestation (n = 39) (Johnson et al. 2000), where the peak systolic pressure trendline was extended to 30 + 2 wks + days gestation and the diastolic LV pressures were assumed to indicate peak LA pressures, and from Devore et al. for stroke volume measurements, ranging from 20 to 40 weeks gestation (n = 200) (Devore et al. 2019). Healthy fetal heart myofiber stresses had not been reported in the literature. We therefore estimated healthy fetal LV myofiber stress via image-based simulations of 6 healthy fetal hearts, as explained in Supplementary Material Section 6.0, where we found that myofiber stresses were 12.19 ± 2.62 kPa and appeared to be invariant with age (Supplementary Fig. S5).

Generally, the reduction in AV dissipative coefficient (from the right of each plot to the left) produced substantial reductions in AV velocity (Fig. 4A–D) and LV pressure (Fig. 4M–P), due to the relief of the stenotic flow and LV fluid congestion, but the extent of the reduction was dependent on how high the pre-FAV AV velocity was. For example, for Patient 2, the initial AV velocity and LV pressure were only modestly high, and thus, the reduction was not as drastic. FAV also generally decreased MVr velocity (Fig. 4E–H), due to more AV outflow after FAV, but the effects on MVr were modest compared to those on AV velocity and LV pressure. Along with the improved AV outflow, stroke volumes substantially improved (Fig. 4I–L), and along with the reduction in LV pressure, myofiber stresses were substantially reduced (Fig. 4U–X). Again, the changes with FAV depended on the initial disease severity. Thus, overall, substantial restoration of LV biomechanics and function toward the healthy state could be achieved with FAV, if stenosis relief was robust, pointing to the robust benefits that could be brought by FAV.

The effects of FAV on LA pressure were more complex (Fig. 4Q–T) and depended on the severity of AVr after FAV. If there was no AVr, decreased AV dissipative coefficient generally led to depressurization of the LA. However, with AVr, the LA pressure did not depressurize as effectively as when there was no AVr present in the vFAV simulations, as end diastolic pressure of the LV was higher, which was then passed back to the LA. Here, we assumed that AVr severity was dependent on the extent of stenosis relief (AVr valve dissipative coefficient was a fixed fraction of that of AV forward flow), which is a likely scenario as a more robust valvuloplasty tended to induce more valve damage, therefore, the effect of AVr preventing LA depressurization was more obvious at lower AV dissipative coefficients, which is demonstrated in Fig. 4Q–T (toward the left side of the plots, where there was more stenosis relief). For scenarios with strong MVr and high MVr velocities, this above effect of AVr on LA pressure was weak (Patient 1 and 2), and the difference between AVr scenarios was not too far from the no AVr scenario, as the strong MVr flow caused LA pressure buildup even without AVr present. Conversely, for patients with lower MVr velocities (Patients 3 and 4), the presence of AVr made a substantial difference to LA pressure. Thus, it was likely that in many cases, the acute effect of FAV would not include depressurization of the LA.

The presence of AVr also had significant effects on LV pressure and stroke volume. Regurgitation further elevated stroke volume beyond the elevation brought by stenosis relief (Fig. 4I–L). Complete relief of stenosis without AVr brought stroke volume up by 12–190%, while complete relief of stenosis with high AVr increased stroke volume by 20–633%. While the improvements to stroke volume did not correspond directly to the same improvement in aortic output, as much of the stroke volume was MVr into the LA, it nonetheless improved the motion of the LV and increased deformational stimuli. Given that stroke volumes of CAS-eHLHS can be very low pre-FAV, as shown in Fig. 4I–L, this additional motion and deformational stimuli provided could have been useful to encourage growth response of the LV to avoid a UV birth outcome. As for LV pressure, the effect of AVr on elevating LV pressure was greater if the baseline LV pressure was low, like that observed in Patients 3 and 4, and was milder if the baseline LV pressure was high, like that observed in Patients 1 and 2.

### Sensitivity analysis of vFAV

The gray dashed lines presented in Fig. 4A–L marked the echocardiographic measurements from follow-up scans of the same patients, which were taken very shortly after FAV. Results from the vFAV simulations (in red, yellow and gray), however, did not always intersect with this post-FAV measurement (gray dashed lines). For AV forward flow velocity in Patient 2 (Fig. 4B), and MVr velocity in Patients 2 and 3 (Fig. 4F, G), an intersection was not found, suggesting that the range of post-FAV scenarios simulated did not match the actual post-FAV situation. Since the vFAV emulated only changes to the AV dissipative coefficients with AVr, without a change to other physiology, such as contractility and peripheral vascular resistances, the mismatch in some cases suggested that there were other physiological changes in addition to the changes to AV dissipative coefficients and presence of AVr. For example, it was likely that Patient 2’s myocardial contractility had increased post-FAV, such that the post-FAV AV velocity was higher than those predicted by vFAV simulations.

**Fig. 5:**
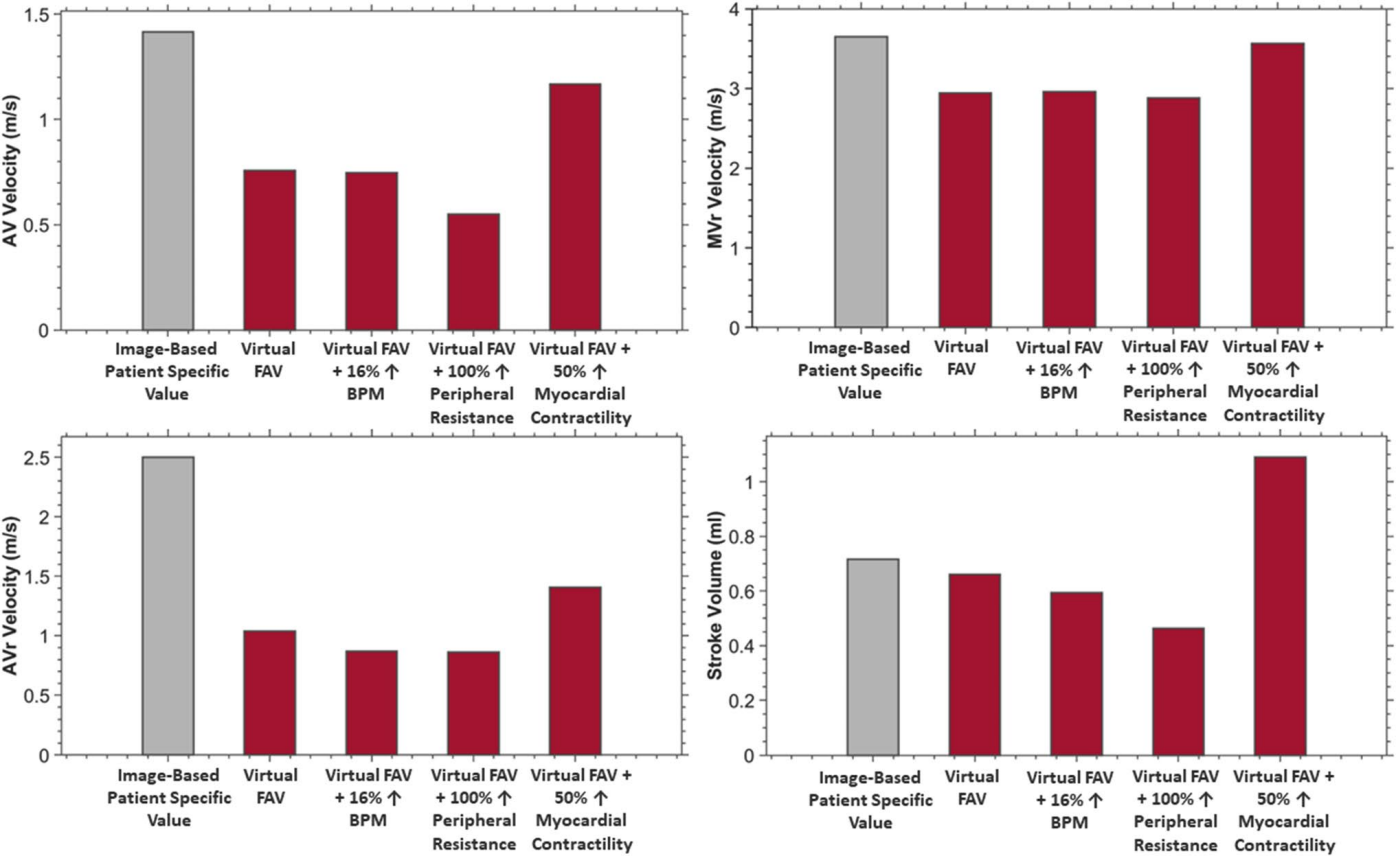
Sensitivity analysis on how physiological changes to heart rate, peripheral vascular resistance and heart rate affected the valve velocities and stroke volume of the post-FAV fetal heart for Patient 2. Red bars correspond to vFAV simulations assuming high AVr and moderate stenosis reduction by FAV, with and without such physiological changes. The gray bar plots the actual post-FAV echo measurement value for Patient 2

We thus performed a sensitivity analysis, to understand how certain physiological changes after FAV impacted the fetal heart, to better understand what were the physiological factors that could account for the mismatch (Fig. 5). We conducted this for Patient 2, as this was the patient with the worst match between vFAV simulations and actual post-FAV measurements. Here, we used the vFAV simulation that had a moderate reduction in AV stenosis and high AVr, as the baseline simulated case, and explored how an altered heart rate, increased peripheral vascular resistance, and increased contractility affected cardiac behavior.

In our cohort of 44 FAV cases, retrospective analysis of pre- to post-FAV beats per minute (BPM) showed little overall change (1.77 ± 8.23% change from 0.65 ± 5.82 BPM at pre-FAV), as shown in Supplementary Fig. S4, however, was nonetheless investigated to assess the impact of BPM. A simulated two standard deviations or 16.46% increase in heart rate (BPM) during the vFAV simulation sensitivity analysis yielded little change to AV and MVr velocities but caused mild decreases in AVr velocity and stroke volume (Fig. 5). Furthermore, a 100% increase in systemic vascular resistance, which meant doubling the peripheral resistive components of the lumped parameter model, caused a decrease in AV and AVr velocity and stroke volume but did not affect MVr velocity (Fig. 5). Finally, when myocardial contractility was increased from 21.91 to 32.87 kPa (a 50% increase), which still remained lower than Racca et al.’s measurements of 49.9 ± 9.3 kPa for an 18.57 weeks gestation healthy fetal LV (Racca et al. 2016), it led to a substantial increase in AV, AVr and MVr velocities and stroke volume (Fig. 5).

Due to the small magnitudes of effects, heart rate was unlikely able to explain the mismatch between vFAV simulation and post-FAV measurements. Further to this, Patient 2’s BPM did not change from pre- to post-FAV, measuring at 133 BPM during both scans, further supporting the unlikeliness of BPM being able to explain the mismatch that was observed between vFAV simulation and post-FAV measurements. Contractility, however, had large effects and increased all 4 measurements in Fig. 5 closer to the actual post-FAV measurements. An increase in contractility was thus a likely factor that could have explained the mismatch. However, increasing contractility would have elevated stroke volume beyond the actual post-FAV measurements. It was thus likely that an increase in peripheral resistance accompanied the increase in contractility to bring stroke volume down.

### Patient-specific post-FAV biomechanics

**Fig. 6:**
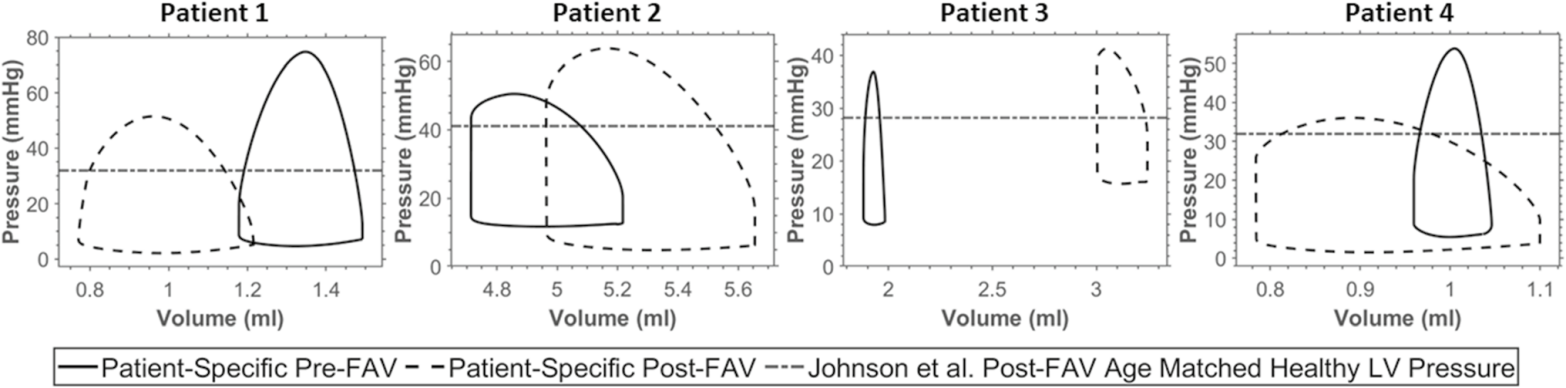
Pre-FAV and post-FAV PV loops, extracted from patient-specific modeling methods, compared to published peak systolic LV pressure measurements of age-matched (to post-FAV age) healthy fetal hearts (Johnson et al. 2000)

**Fig. 7:**
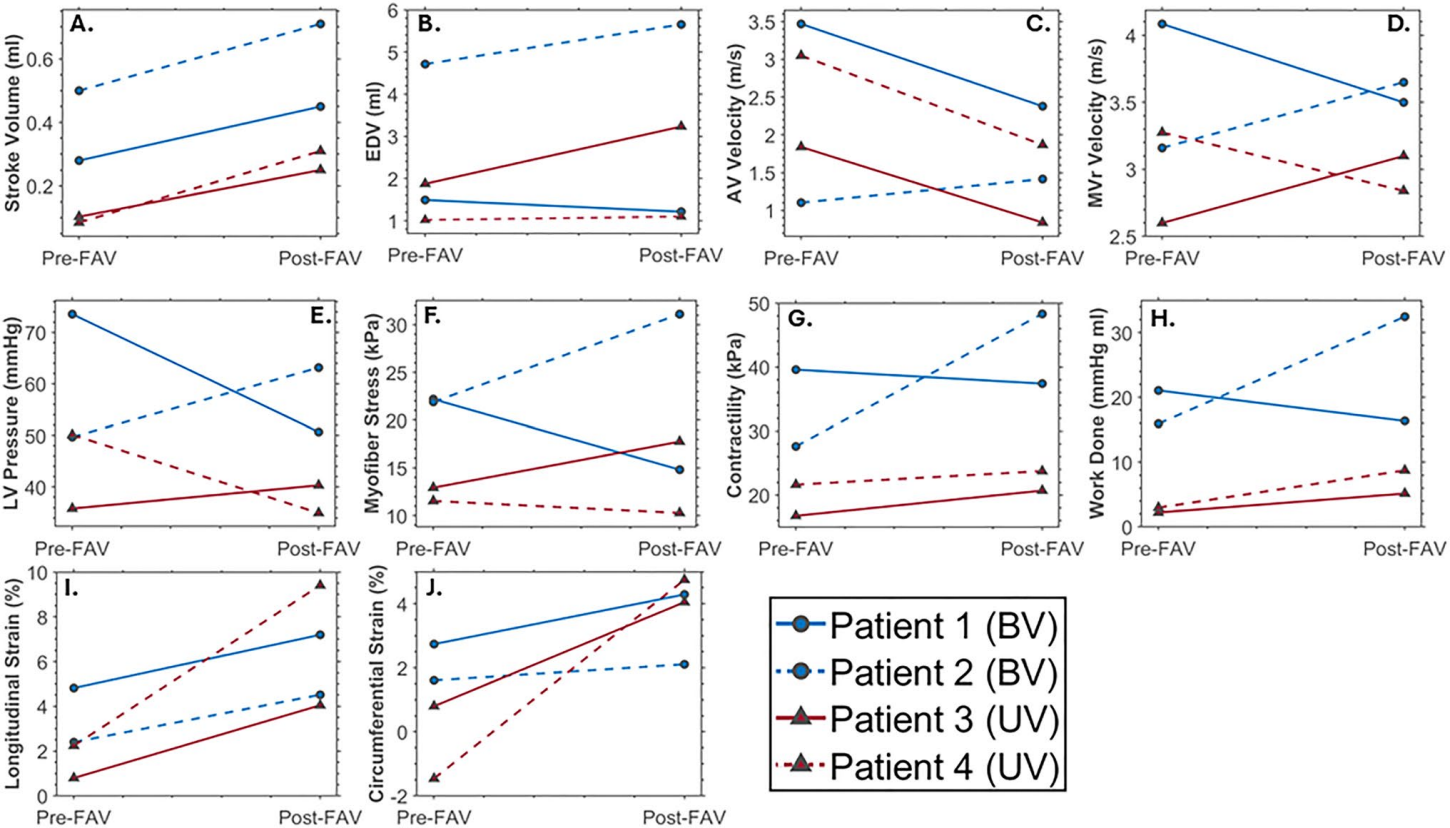
Pre-FAV versus post-FAV fetal LV characteristics. Stroke volume, end diastolic volume (EDV), valve velocities, and strains were measured via echo, while LV pressure, myofiber stress, contractility, and work done by the LV were back-computed via our iterative patient-specific FE computational modeling

To better understand the actual patient-specific post-FAV biomechanics, we conducted image-based simulations on the same patients using their post-FAV scans and compared the outputs to the corresponding pre-FAV values (Figs. 6 and 7). The post-FAV PV loops compared to pre-FAV loops, detailed in Fig. 6, demonstrated that FAV had substantially increased stroke volume, however, interestingly, peak systolic pressure had not always reduced from its high diseased magnitude, despite the reduction in AV stenosis, as pressure was reduced in Patients 1 and 4, but was elevated in Patients 2 and 3. The variation in pressure change results was likely due to a combination of effects, where stenosis relief reduced LV pressure, as seen in the vFAV scenarios and increased contractile forces elevate pressure, and the degree to which each occurred was patient dependent. For all cases, the post-FAV peak systolic LV pressure remained elevated and did not come down to the healthy LV level, depicted by the horizontal dotted line in Fig. 6.

The back-computed valve dissipative coefficients for antegrade AV and MV flow output from the pre- and post-FAV models have been documented in Table 3. To enable a better appreciation of the aortic stenosis severity, the log ratio of the diseased versus healthy dissipative coefficients has also been presented. These results demonstrated that FAV typically created substantial relief in AV stenosis and substantially reduced the AV dissipative coefficient, but flow dissipative coefficients remained substantially higher than those of the age-matched healthy fetal hearts. Patient 2 was an exception, where the initial AV dissipative coefficient was not exceptionally high, and FAV only decreased the AV dissipative coefficient slightly. The range of AV dissipative coefficients considered by our vFAV simulations is also given in Table 3, and it could be observed that they encompassed the actual post-FAV dissipative coefficient, which demonstrated that our vFAV covered the appropriate stenosis relief scenarios.

The post-FAV functional and biomechanics characteristics compared to their corresponding pre-FAV values are shown in Fig. 7, the values of which are provided in Supplementary Table S4. As with Fig. 6, we could observe here that FAV resulted in an increase in stroke volume (Fig. 7A). Consequent to larger stroke volume, longitudinal strain (Fig. 7I) and circumferential strain (Fig. 7J) increased as well for all patients, which demonstrated the ability of FAV to enable greater myocardial deformations, which could be important for healthier subsequent cardiac development. For the remaining characteristics investigated, including EDV, AV velocity, MVr velocity, LV pressure, peak myofiber stress, myocardial contractility and work done (Fig. 7B–H), pre- to post-FAV trends were not uniform across patients.

As discussed above, LV pressure (Fig. 7E) did not always reduce and likely depended on contractility and the extent of AV stenosis relief. For example, a substantial post-FAV increase in myocardial contractile forces was found for Patient 2 (Fig. 7G), and a consequent post-FAV elevation in LV pressure was observed. However, this elevation in LV pressure in Patients 2 and 3 was likely responsible for the post-FAV elevation in their myocardial stresses, and in MVr velocities, as higher LV pressure induced higher wall stresses and drove stronger MVr. In the same way, despite the reduction in AV flow dissipative coefficient, AV velocity did not always reduce and was elevated post-FAV for Patient 2. Again, this could have been attributed to its post-FAV elevation in contractility. While FAV led to reduced AV stenosis brought about by a reduced AV velocity toward lower healthy values, elevated contractility and increased LV outflow could then have increased AV velocities, and the net effect could by an increased AV velocity, like that observed in Patient 2. As for the pre- to post-FAV trends observed for EDV, they are likely related to LV pressure, as increased LV pressures, could have led to LV distention and influenced the severity of AV and MVr, which would have then determined the extent of diastolic filling.

Among the 4 cases, there was substantial variability, with varying extent of changes to myocardial contractility, LV pressure, AV velocities and other characteristics. The individuality of each patients’ results highlighted the complex nature of CAS-eHLHS biomechanics, and the impact FAV had.

## Discussion

In this study, we performed vFAV, to comprehend the effect of the procedure on the biomechanics and function of the fetal LV. We studied this with two approaches. First, we performed simulations based on pre-FAV echo images and then performed vFAV by imposing a reduction in AV flow and introducing AVr. This was an idealized approach, assuming that only the AV was affected by FAV and that there were no other physiological responses, which served the purpose of elucidating the mechanistic effects of AV stenosis relief alone. Secondly, we performed simulations based on post-FAV images, to compare them to pre-FAV simulations, to understand the overall changes.

Diseased CAS-eHLHS LVs typically had high pressures and tissue stresses, and very low myocardial strains and stroke volume. They further had high atrial pressure and an enlarged LA (Pickard et al. 2020; Tulzer et al. 2022). This unusual biomechanical environment likely prevented normal development of the LV and LA. Our vFAV simulations showed that by relieving AV stenosis, FAV depressurized the LV, reduced abnormally high AV velocities, reduced MVr severity, increased stroke volume, reduced myocardium tissue stresses, and, if AVr was not present, reduced LA pressure as well. All these represented partial normalizations of cardiac function and biomechanics, enabling lower stresses and increased deformational stimuli of myocardial tissue, and were thus likely beneficial for the subsequent development of the heart, to avoid progression to a UV birth outcome.

However, a complexity in this investigation was the presence of AVr, which was very common after FAV (Bradford et al. 2022). vFAV simulations showed that the presence of AVr dampened the ability of the LV and LA to depressurize, and dependent on regurgitation severity, could even increase LA pressure. This was because AVr brought the higher aortic pressure into the LV, through regurgitation during diastole, and the higher pressure was passed on to the LA. In our previous fluid dynamics simulation study, we further showed that AVr resulted in excessive energy losses and high cardiac workload (Wong et al. 2023), which reduced the restoration of cardiac flow function. However, AVr promoted higher stroke volume and higher strains of myocardial tissues, and previous authors (Arzt et al. 2011; Wong et al. 2023) speculated that this higher deformational stimulus was beneficial to subsequent cardiac development. It was, however, noteworthy that our current study was limited to the acute outcomes of FAV. Later in gestation, AVr often improved or resolved over subsequent gestational development (Marshall et al. 2005), which in those circumstance could lead to further normalization of function and biomechanics, as demonstrated in the vFAV with no AVr scenarios.

Our investigations of post-FAV scans provided validation that the stroke volume and myocardial strains indeed increased, while our FE simulations based on post-FAV scans confirmed that FAV indeed reduced AV valve dissipative coefficient and relieved the stenosis. However, our results suggested that stenosis was not fully relieved. This corroborated with our previous image-based fluid dynamics studies, where we estimated that although the AV effective orifice area doubled after FAV, it remained substantially lower than (about one-fifth that of) that of a healthy fetal heart (Wong et al. 2023). The increase in circumferential and longitudinal strains agreed with previous findings by Ishii et al. which showed FAV to promote increased myocardial strains (Ishii et al. 2012).

Our studies of post-FAV scans and simulations also revealed that some fetal LV cases did not experience a great reduction in AV velocity or LV pressure, despite relief of the stenosis and LV fluid congestion. Clinically, we have often used AV velocity and Bernoulli’s equation to estimate valvular pressure gradient, and a failure to reduce AV velocity would have falsely implied that FAV did not relieve the stenosis. Our investigations, however, revealed that stenosis was indeed relieved for all cases, but there were likely other physiological responses, such as elevated myocardial contractile forces and peripheral vascular resistance, that negated the reduction of AV velocity and LV pressure. This complex interplay between various physiological features served as a reminder that we should not use simple single measures to assess stenosis severity during FAV, and a complete image-based FE model might be more appropriate. It seemed reasonable to expect some LVs to have elevated contractility after FAV, which could be induced by the increased deformational stimuli of the LV and an end-diastolic distension of the LV due to AVr, in line with the Frank–Starling mechanism. In our results, myocardial contractility increased across FAV for all patients, except Patient 1, whose myocardial contractility in fact maintained at a similar level despite a slight decrease (Fig. 7G). Our sensitivity analysis showed that contractility changes had a significant effect on cardiac function and biomechanics. Contractility changes across FAV were thus an important feature to consider. In terms of peripheral vascular resistance changes, our sensitivity analysis also suggested that this could occur after FAV, however, this physiological change had a milder effect.

Our modeling results demonstrated that patient-specific modeling can enable a detailed investigation of the complex biomechanical and physiological effects of FAV and was useful in capturing the individuality of each patient, addressing the large patient-to-patient variability. However, we found that the computational prediction of acute outcomes of FAV remained difficult, because it was not currently possible to predict how much of the stenosis would be relieved by FAV and how much (if any) the fetal heart contractility would alter, which is important as stenosis severity and contractility were shown to be very influential parameters with significant effects on simulation outcomes. However, it might be the case that with a much larger sample size, we can obtain the statistical distribution of these physiological changes after FAV, to help with computational predictions.

Finally, comparing features of patients that went on to be BV (Patient 1 and 2) or UV (Patient 3 and 4) post-FAV, our results showed that UV patients LVs remained relatively low pressure compared to the BV cases post-FAV, and they also had lower stroke volume, work done and myocardial contractility. This appeared to support the hypothesis that the biomechanically “weaker” fetal hearts were more likely to progress to a UV outcome, but the sample size here is low and cannot provide enough evidence to test this hypothesis. However, clinical estimations of LV pressure were made by Tulzer et al. and McElhinney et al., who concluded that the higher-pressure pre-FAV LVs have a greater chance of progressing to a BV outcome post-FAV (McElhinney et al. 2009; Tulzer et al. 2022). This corroborated the findings of our study.

Future work is warranted, for several reasons. Firstly, the underlying reasons for the later resolution of AVr post-FAV remains unclear, despite it being a positive event. Conducting a longitudinal study would help elucidate the biomechanical changes occurring later in gestation among post-FAV patients and help comprehend how these changes impact the presence of AVr. Secondly, our study highlighted the necessity of patient specific post-FAV modeling for assessing acute outcomes. However, to establish broader trends among post-FAV patients, a larger, multi-center study is imperative. Such a study could identify functional trends post-FAV, which could support postnatal surgical planning. However, to achieve such a large-sample study, our iterative FE simulations need to be sped up via deep learning.

There were several limitations of this study. First, our biomechanics simulations required idealizations. For example, we assumed the same myocardial helix angle configuration for all LVs and applied the same myocardial stiffness across all cases. Helix angle configuration could vary from person to person, while myocardial stiffness would likely change across gestational ages, and there could be errors in the computed biomechanics and function. Further, our modeling of AVr and MVr was simplified, to a constant valve dissipative coefficient across diastole and systole, respectively. Third, fetal echo tended to have high levels of noise, which could have resulted in inaccuracies in our image-based computational modeling. This might account for some of the mismatch between vFAV and actual post-FAV measurements and simulations. Fourth, in our patient-specific optimization to match clinical measurements, some percentage errors remained high. Additional complexity in the FE and lumped-parameter models might be needed to reduce the percentage errors. Given the monotonic relationship between the parameter optimized for (valve dissipative coefficients or contractile tension) and the parameter that was matched (valve pressure gradients and stroke volume) and given that convergence on a solution is usually within a few iterations, solution uniqueness seemed likely, but we do not have formal proof of uniqueness. Fifth, in our sensitivity analysis, we had chosen to investigate a limited number of factors for investigation only based on an educated guess, and there could be other unknown factors capable of affecting results.

## Conclusion

We conducted image-based computational modeling to understand the biomechanical impact of FAV intervention. Our vFAV simulations showed that FAV likely improved LV functionality, by depressurizing the LV and LA, reducing AV velocity and MVr velocity, reducing myofiber stress, and increasing stroke volume. The presence of AVr moderately inhibited LV depressurization and prevented LA depressurization; however, it provided substantial augmentation of stroke volume and myocardial strains, which could have provided beneficial stimuli to further cardiac development. Through comparisons of vFAV to actual post-FAV images and simulations, we found that cardiac contractility had likely increased after FAV, which had unexpectedly increased AV forward velocities and LV pressure, contrary to the expectation that stenosis should have decreased them. Post-FAV simulations demonstrated substantial stenosis relief, but AV dissipative coefficient remained quite different from those of healthy hearts.

## Supplementary Information

Below is the link to the electronic supplementary material.

Supplementary file1 (DOCX 1983 KB)

## Abbreviations

AV: Aortic valve
AVr: Aortic valve regurgitation
BV: Biventricular
BPM: Beats per minute
CAS-eHLHS: Critical aortic stenosis with evolving hypoplastic left heart syndrome
Dil: Balloon dilation of the aortic valve
EDV: End diastolic volume
eHLHS: Evolving hypoplastic left heart syndrome
FAV: Fetal aortic valvuloplasty intervention
FE: Finite element
LA: Left atrium
LV: Left ventricle
MV: Mitral valve
MVr: Mitral valve regurgitation
NW: Norwood procedure
PV: Pressure volume
RK: Ross-Kono procedure
RV: Right ventricle
UV: Univentricular
vFAV: Virtual fetal aortic valvuloplasty intervention
Wks + days: Weeks and days
