# Supplementary material for: Myocardial biomechanical effects of fetal aortic valvuloplasty
Source: Biomech Model Mechanobiol. 2024 Apr 29;23(5):1433–48. doi: 10.1007/s10237-024-01848-0 (PMC11436463; doi:10.1007/s10237-024-01848-0)
Supplement: Supplementary file 1 — Supplementary file1 (DOCX 1983 KB) [file 10237_2024_1848_MOESM1_ESM.docx]

**Supplementary Material**

**1.0 Demonstration of Cardiac Motion Estimation Pre- and Post-Fetal Aortic Valvuloplasty (FAV) Intervention and Image-Based Strain Calculations**

Demonstration of each patients cardiac motion tracking, pre- and post-FAV intervention, at end diastolic volume (EDV) and end systolic volume (ESV), Figure S1. The inner wall was extracted to measure LV volume variation over the cardiac cycle.

**
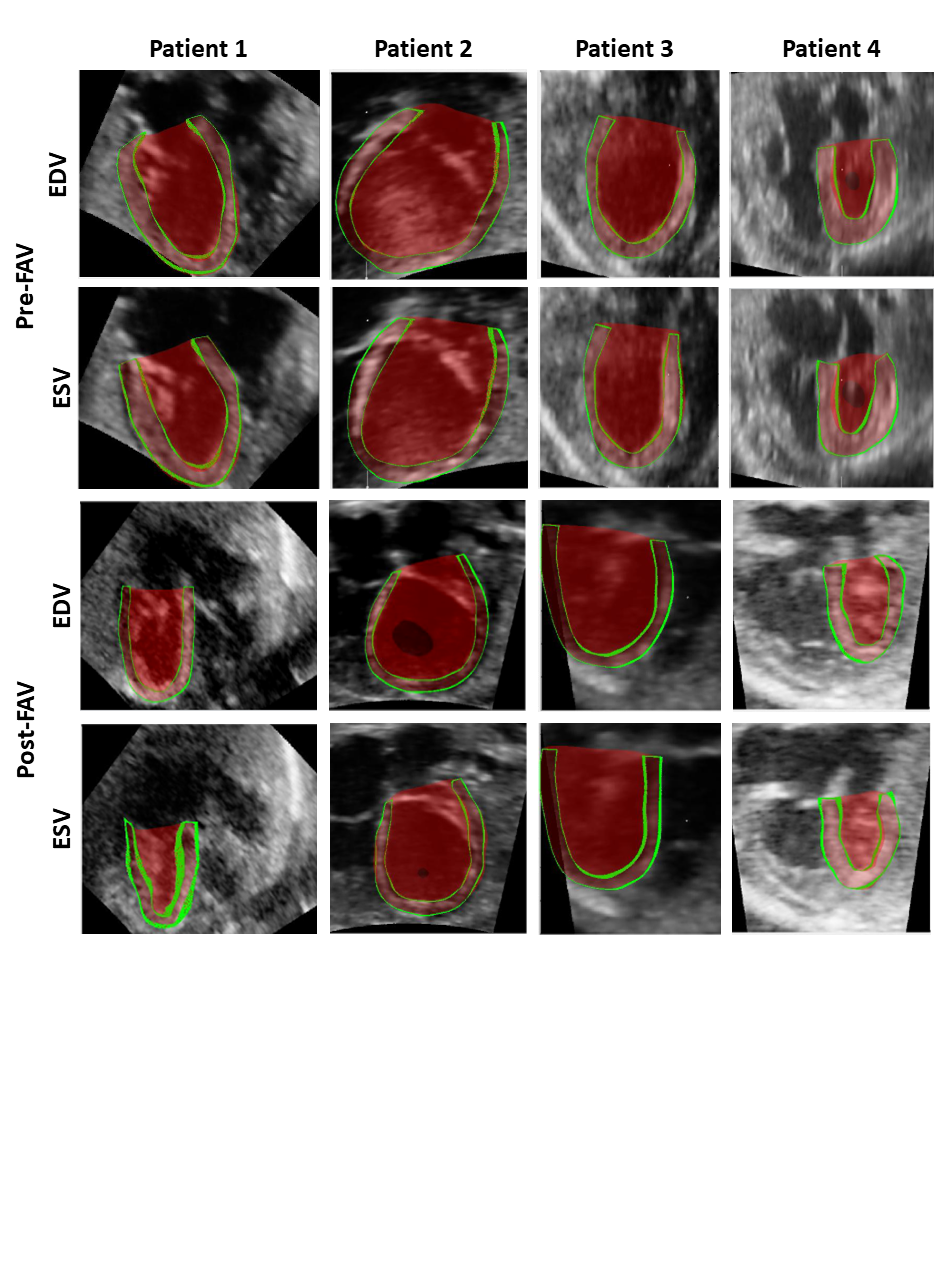
**

**Figure S1.** Reconstructed geometries superimposed onto associated echo image, green outline highlighting geometry proportion close to current image plane, for all patients pre- and post-FAV at EDV and ESV time points.

Cardiac motion estimation results were utilised to compute image-based strains in the circumferential and longitudinal direction. Figure S2A has been provided to demonstrate the averaging of the epicardial and endocardial boundaries, which computed the mid-wall points for Patient 2 and Figure S2B shows how the circumferential and longitudinal strains were visualized.


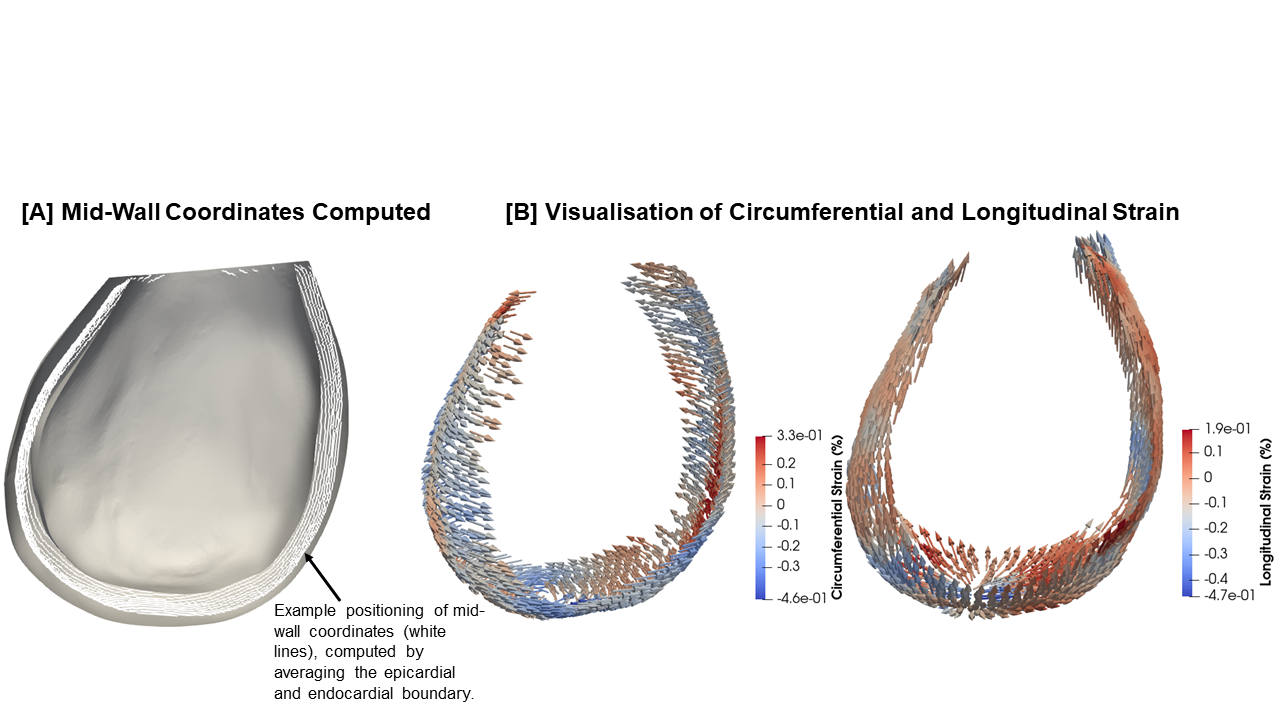


**Figure S2:** Myocardial strain calculation and visulisation.

**2.0 Further Finite Element (FE) Modelling Parameters**

The cardiac cycle length was extracted from each patients scans pre- and post-FAV and then converted to beats per minute (BPM), where time to peak tension ($t_{0}$) was calculated, based on Mulieri et al.’s derivation (Mulieri et al., 1992), with all values documented in Table S1.

**Table S1.** Patient specific beats per minute (BPM) and the time to peak tension ($t_{0}$) derived from a published correlation (Mulieri et al., 1992).

|  | **Pre-FAV** | | **Post-FAV** | |
| --- | --- | --- | --- | --- |
|  | BPM | Time to peak tension - $t_{0}$ (ms) | BPM | Time to peak tension - $t_{0}$ (ms) |
| Patient 1 | 150 | 130 | 139 | 135 |
| Patient 2 | 133 | 137 | 133 | 137 |
| Patient 3 | 147 | 131 | 157 | 128 |
| Patient 4 | 153 | 129 | 148 | 131 |

**3.0 Lumped Parameter Model**

The components described in the lumped parameter model and their connections are shown in Figure S3, the schematic has been adopted from previous work (Pennati et al., 1997).

**
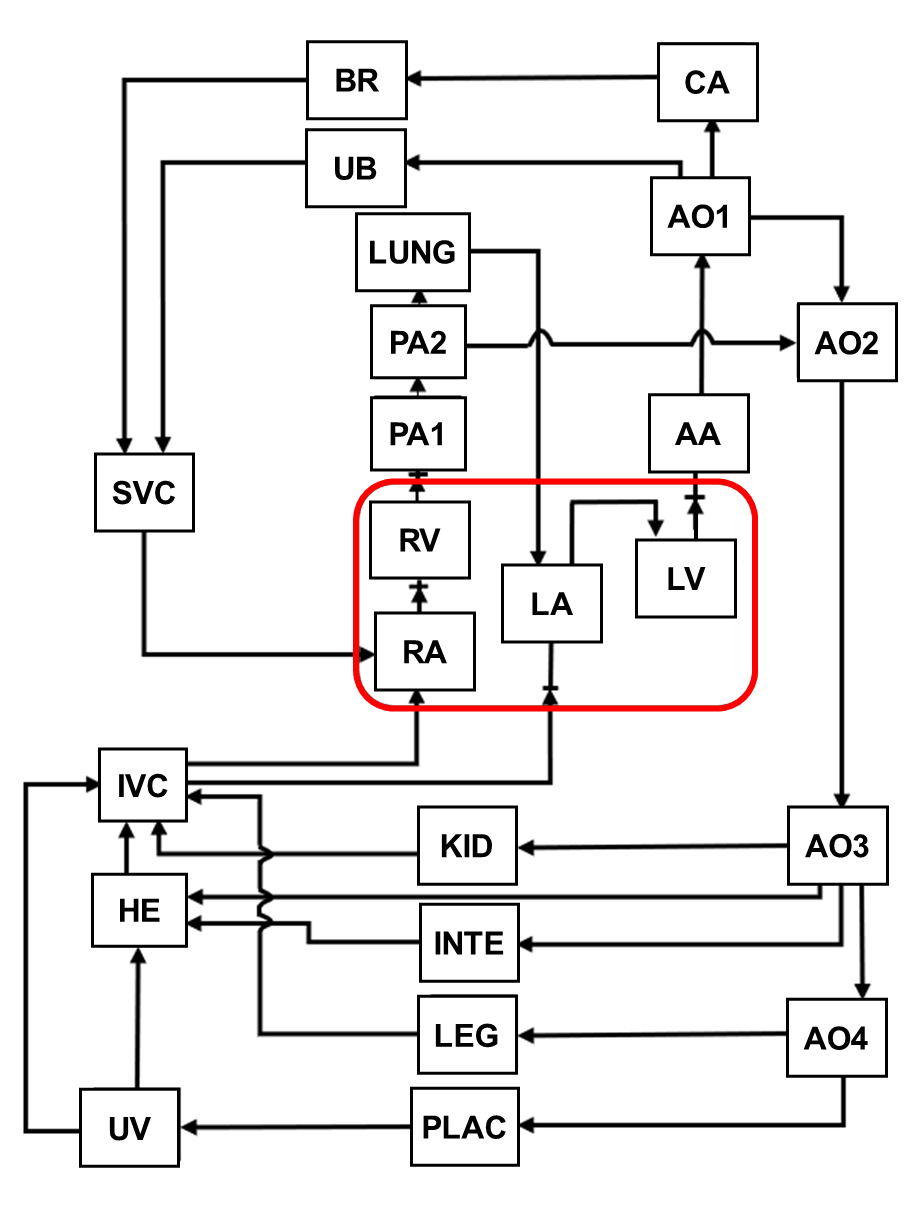
**

**Figure S3.** Schematic of lumped parameter model, adopted from previous work (Pennati et al., 1997). Note, AA: ascending aorta, AO1: aortic arch, AO2: thoracic descending aorta, AO3: abdominal descending aorta, AO4: femoral bifurcation, BR: brain, CA: cerebral arteries, HE: liver, INTE: intestinal circulation, IVC: inferior vena cava, KID: kidney, LA: left atrium, LEG: lower limbs, LUNG: lungs, LV: left ventricle, PA1: main pulmonary artery, PA2: pulmonary arteries, PLAC: placenta, RA: right atrium, RV: right ventricle, SVC: superior vena cava, UB: upper body, UV: umbilical vein.

The original lumped parameter model was calibrated according to the fetal lamb and where possible fetal human data (Pennati et al., 1997), for a 38 week gestation period. Age scalability of the lumped parameter model was introduced in later work (Pennati. & Fumero., 2000), by scaling the 38 week gestation lumped parameter model based on the principal that any biological variable can be related to its body size via the following allometric equation,

| $Y_{WG}=Y_{38}\cdot\left( \frac{W_{WG}}{W_{38}} \right)^{b},$ | Equation S1 |
| --- | --- |

where $Y_{WG}$ was the value at the week gestation (WG) required, $Y_{38}$ was the reference value, relating to the 38 WG time point, $W_{WG}$ was the fetal weight at the WG required, $W_{38}$ was the fetal weight at the reference value of 38 WG and $b$ was the scaling factor.

Using the allometric formulation previously described (Equation S1) Pennati et al. derived the following equations for scaling all circulatory components of the lumped parameter model dependent on gestational age. Where the resistance at the age of interest, $R_{WG}$, was scaled based on the reference resistance value, $R_{38}$, with a scaling factor of $-1$, as shown,

| $R_{WG}=R_{38}\cdot\left( \frac{W_{WG}}{W_{38}} \right)^{-1}.$ | Equation S2 |
| --- | --- |

The same principles followed for the dissipative terms in the lumped parameter model at the fetal age being investigated, $D_{WG}$, with a scaling factor of $-1.33$, which gave,

| $D_{WG}=D_{38}\cdot\left( \frac{W_{WG}}{W_{38}} \right)^{-1.33}.$ | Equation S3 |
| --- | --- |

The inductance at the specific fetal age required, $L_{WG}$, was scaled by a factor of $-0.33$, which gave,

| $L_{WG}=L_{38}\cdot\left( \frac{W_{WG}}{W_{38}} \right)^{-0.33}.$ | Equation S4 |
| --- | --- |

Finally, capacitance at the specific fetal age required, $C_{WG}$, was scaled by a factor of $1.33$, which gave,

| $C_{WG}=C_{38}\cdot\left( \frac{W_{WG}}{W_{38}} \right)^{1.33}.$ | Equation S5 |
| --- | --- |

During the derivation of the original scaled lumped parameter model detailed above, some assumptions were made which have now been disproven. It was assumed that blood viscosity remained constant with gestational age, which has since been disproven (Jopling et al., 2009; Kwon et al., 2008), as hematocrit percentage was shown to change with gestational age (Jopling et al., 2009) and changes in hematocrit levels have been shown to vary blood viscosity (Kwon et al., 2008). It was also assumed that the relationship between Young’s Modulus and vessel thickness remained constant across gestational development, this is now believed to be incorrect, as Jacot et al. showed that neonatal mice LVs Young’s Modulus was approximately 3 times greater than at embryonic stages (Jacot et al., 2010) and from clinical measurements LV wall thickness was shown to increase with gestation age (Daimei et al., 2014). Also, at the time of lumped parameter model development, fetal LV intracardiac pressure measurements were not available, the later published measurements (Johnson et al., 2000) were useful in this study to help establish the healthy baselines. When fetal LV pressure and aortic pulse pressure measured in literature (Johnson et al., 2000; Versmold et al., 1981) were compared to outputs from the original lumped parameter model a root mean square error (RMSE) of 13.93 and 13.64 respectively was calculated. Therefore, minimal but uniform changes were applied to the model to improve the match with literature-based pressure measurements (Johnson et al., 2000; Versmold et al., 1981), whilst limiting disruption to the established relationships derived previously (Pennati et al., 1997; Pennati. & Fumero., 2000). Through an iterative approach an additional scaling value was applied to the resistance, $R_{scale}$, described in Equation S6, and applied in Equation S7. Following this a constant scaling factor of 0.22, represented by $C_{scale}$ in Equation S8 was applied to the capacitance scaling. The application of such scaling factors improved the RMSE from 13.93 to 2.01 for peak LV pressure and 13.64 to 1.05 for aortic pulse pressure.

| $R_{scale}=0.0002567W_{WG}^{3}-0.01759W_{WG}^{2}+0.4228W_{WG}-3.0509$ | Equation S6 |
| --- | --- |

| $R_{WG}= {R_{scale}\times R}_{38}\left( \frac{W_{WG}}{W_{38}} \right)^{-1}.$ | Equation S7 |
| --- | --- |

| $C_{WG}= C_{scale}\times C_{38}\left( \frac{W_{WG}}{W_{38}} \right)^{1}.$ | Equation S8 |
| --- | --- |

1. **Retrospective Data Collection for Heart Rate Change Pre- to Post-FAV Intervention**

Data was retrospectively collected for aortic valve regurgitation (AVr) velocity, post FAV, for n=44, and change in beats per minute (BPM), from pre- to post-FAV intervention, for n=34 patients, Figure S4. Average AVr velocity post-FAV was 1.92±0.87 m/s and the average BPM increased by 1.77% post-FAV compared to pre-FAV, with a standard deviation of ±8.23.


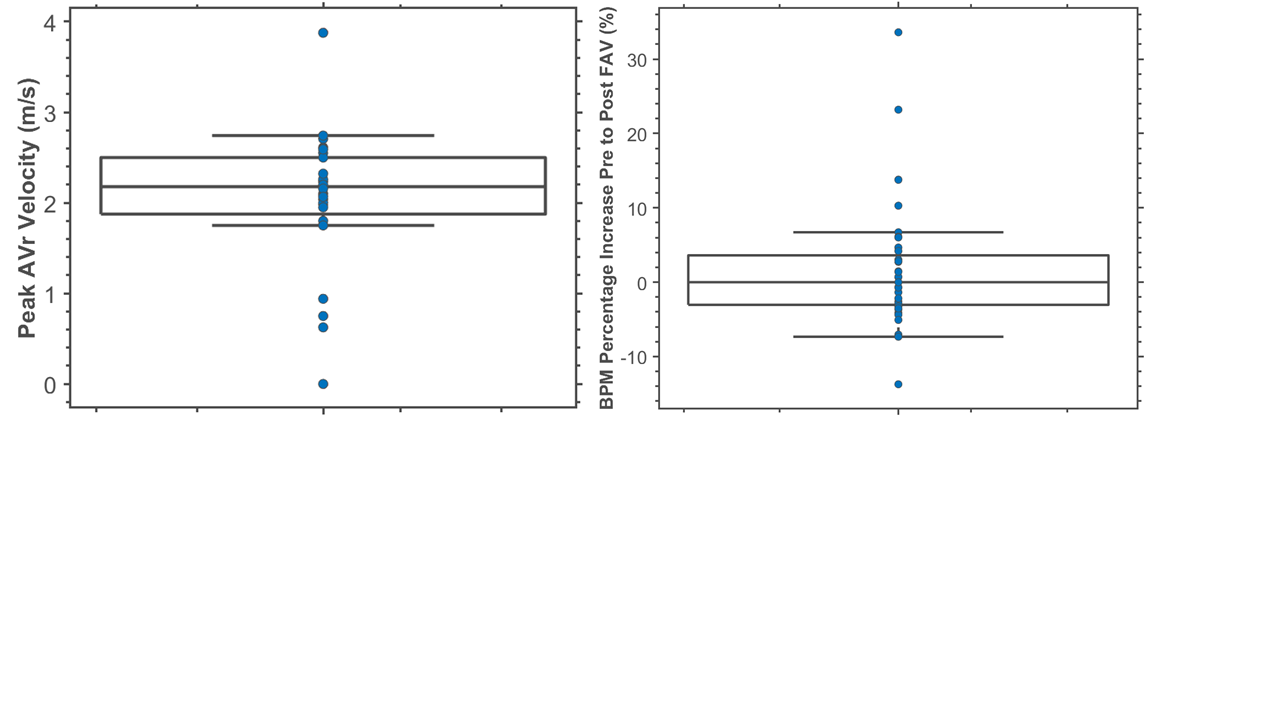


**Figure S4.** Bar plot showing AVr velocity post-FAV (n=44) and percentage increase in BPM, from pre- to post-FAV intervention (n=34).

**5.0 Pre- and Post-FAV Biomechanics Characteristics**

Table S2 and S3 detailed the final match between image and simulated stroke volume, aortic valve (AV) pressure gradient (ΔP), mitral valve (MV) ΔP, AVr ΔP and mitral valve regurgitation (MVr) ΔP, for all patients, for pre- and post-FAV, patient-specific image-based simulations, demonstrating a satisfactory match (Note: AVr ΔP not optimised for in pre-FAV models as was not present). Errors for stroke volume were -0.74±4.57%, while that for valve gradients were -2.14±14.24%. Patient 3s post-FAV MVr ΔP was especially difficult to match, and had an error of 38%, which could be due cumulatively to errors in echo measurements and idealization assumptions of the FE and lumped parameter model.

**Table S2.** Image vs simulation match for all pre-FAV models.

|  | **ID** | **Stroke Volume (ml)** | **AV ΔP (mmHg)** | **MV ΔP (mmHg)** | **MVr ΔP (mmHg)** |
| --- | --- | --- | --- | --- | --- |
| Image | Patient 1 | 0.28 | 48.12 | 3.69 | 66.75 |
| Simulation |  | 0.31 | 47.60 | 3.32 | 65.39 |
| Image | Patient 2 | 0.50 | 4.87 | 1.49 | 39.94 |
| Simulation |  | 0.50 | 5.65 | 2.02 | 36.32 |
| Image | Patient 3 | 0.10 | 13.54 | 27.04 | 27.04 |
| Simulation |  | 0.10 | 13.91 | 27.30 | 27.30 |
| Image | Patient 4 | 0.085 | 37.30 | 42.91 | 42.91 |
| Simulation |  | 0.084 | 39.59 | 42.56 | 42.56 |

**Table S3.** Image vs simulation match for all post-FAV models.

|  | **ID** | **Stroke Volume (ml)** | **AV ΔP (mmHg)** | **MV ΔP (mmHg)** | **AVr ΔP (mmHg)** | **MVr ΔP (mmHg)** |
| --- | --- | --- | --- | --- | --- | --- |
| Image | Patient 1 | 0.45 | 22.66 | 3.62 | 19.36 | 48.96 |
| Simulation |  | 0.45 | 22.77 | 3.91 | 17.38 | 44.52 |
| Image | Patient 2 | 0.71 | 8.02 | 2.54 | 25.00 | 53.40 |
| Simulation |  | 0.69 | 8.35 | 3.17 | 21.49 | 55.43 |
| Image | Patient 3 | 0.25 | 2.86 | 1.69 | 13.99 | 38.44 |
| Simulation |  | 0.24 | 3.01 | 1.67 | 16.40 | 23.81 |
| Image | Patient 4 | 0.31 | 13.96 | 2.15 | 20.43 | 32.21 |
| Simulation |  | 0.32 | 15.39 | 2.77 | 19.40 | 30.96 |

Pre- and post-FAV physiological characteristics from scans and biomechanics characteristics from simulations, as demonstrated in Figure 7, were also given here in Table S4. Age-matched LV pressure for a healthy heart was also given, based on intracardiac pressure measurements by (Johnson et al., 2000).

**Table S4.** Comparison of pre- and post-FAV fetal biomechanics, extracted from the patient specific computational methods.

|  | **Patient 1**  **(BV outcome)** | | **Patient 2**  **(BV outcome)** | | **Patient 3**  **(UV outcome)** | | **Patient 4**  **(UV outcome)** | |
| --- | --- | --- | --- | --- | --- | --- | --- | --- |
|  | Pre-FAV | Post-FAV | Pre-FAV | Post-FAV | Pre-FAV | Post-FAV | Pre-FAV | Post-FAV |
| Stroke Volume (ml) | 0.28 | 0.45 | 0.50 | 0.71 | 0.10 | 0.25 | 0.085 | 0.31 |
| EDV (ml) | 1.49 | 1.22 | 4.71 | 5.65 | 1.88 | 3.23 | 1.02 | 1.10 |
| LV Pressure (mmHg) | 73.55 | 50.69 | 49.72 | 63.15 | 35.83 | 40.34 | 50.14 | 34.96 |
| Age-Matched Healthy Heart LV Pressure (Johnson et al.) | 31.71 | 31.96 | 39.67 | 41.17 | 27.73 | 28.23 | 31.71 | 31.96 |
| Work Done (mmHg ml) | 21.05 | 16.35 | 15.93 | 32.46 | 2.22 | 5.16 | 2.98 | 8.74 |
| Peak Myofiber Stress (kPa) | 22.19 | 14.82 | 21.91 | 31.12 | 12.93 | 17.77 | 11.53 | 10.27 |
| Myocardial Contractility (kPa) | 39.59 | 37.41 | 27.64 | 48.31 | 16.78 | 20.73 | 21.65 | 23.77 |
| Longitudinal Strain (%) | 4.82 | 7.20 | 2.40 | 4.52 | -0.66 | -0.48 | 2.25 | 9.41 |
| Circumferential Strain (%) | 2.74 | 4.29 | 1.61 | 2.11 | 0.80 | 4.05 | -1.46 | 4.75 |

**6.0 Healthy Peak Systolic Myofiber Stress Data**

Healthy peak systolic myofiber stress data was extracted from the patient specific optimization methods (n=6). From Figure S5 there was a very weak negative correlation between peak systolic myofiber stress and gestational age (r=-0.10), the average peak systolic myofiber stress of 12.19 kPa was used as the healthy benchmark.

**
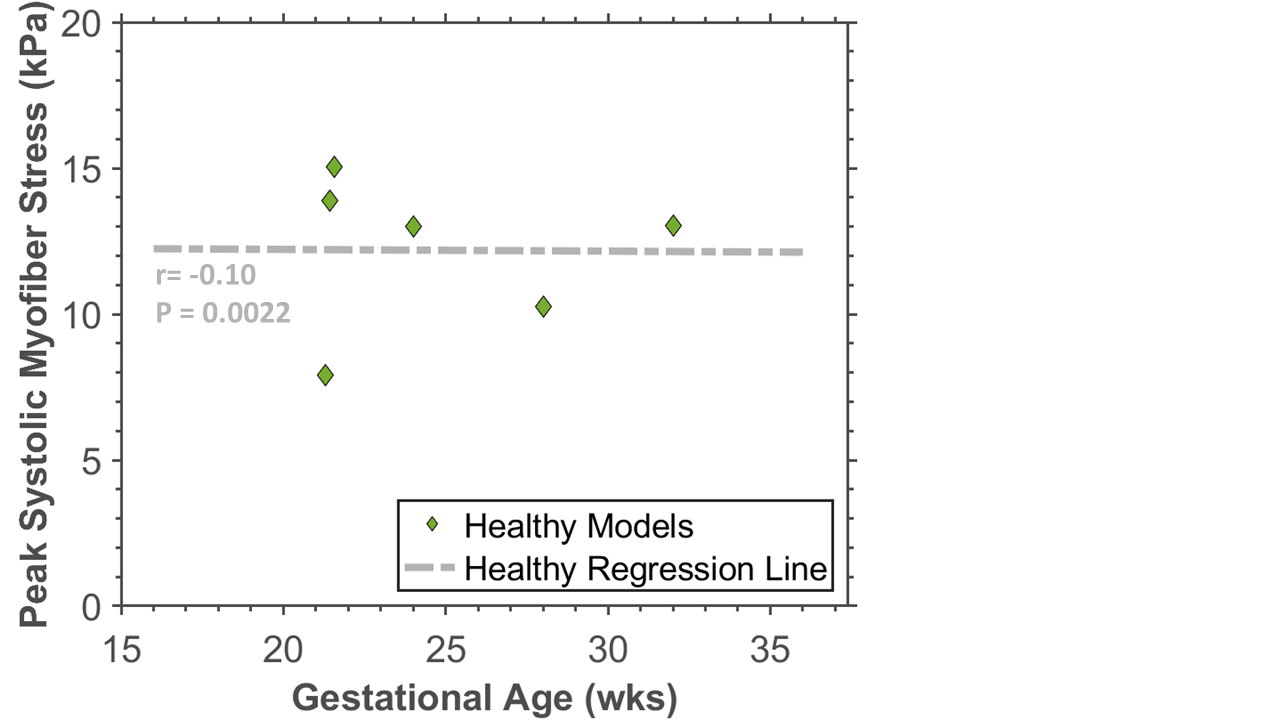
**

***Figure S5.*** *Relationship between peak systolic myofiber stress and gestational age, output from patient specific computational methods.*

**References**

Daimei, T., Devi, D., & Sinam, V. (2014). Difference between the left and right ventricular thickness in fetal heart. *IOSR Journal of Dental and Medical Sciences*, *13*(4), 21–24. https://doi.org/10.9790/0853-13412124

Jacot, J. G., Martin, J. C., & Hunt, D. L. (2010). Mechanobiology of Cardiomyocyte Devlopment. *Journal of Biomechanics*, *43*(1), 1–13. https://doi.org/10.1016/j.jbiomech.2009.09.014.Mechanobiology

Johnson, P., Maxwell, D. J., Tynan, M. J., & Allan, L. D. (2000). Intracardiac pressures in the human fetus. *Heart*, *84*(1), 59–63. https://doi.org/10.1136/heart.84.1.59

Jopling, J., Henry, E., Wiedmeier, S. E., & Christensen, R. D. (2009). Reference ranges for hematocrit and blood hemoglobin concentration during the neonatal period: Data from a multihospital health care system. *Pediatrics*, *123*(2). https://doi.org/10.1542/peds.2008-2654

Kwon, O., Krishnamoorthy, M., Cho, Y. I., Sankovic, J. M., & Banerjee, R. K. (2008). Effect of blood viscosity on oxygen transport in residual stenosed artery following angioplasty. *Journal of Biomechanical Engineering*, *130*(1). https://doi.org/10.1115/1.2838029

Mulieri, L. A., Hasenfuss, G., Leavitt, B., Allen, P. D., & Alpert, N. R. (1992). Altered myocardial force-frequency relation in human heart failure. *Circulation*, *85*(5), 1743–1750. https://doi.org/10.1161/01.CIR.85.5.1743

Pennati, G., Bellotti, M., & Fumero, R. (1997). Mathematical modelling of the human foetal cardiovascular system based on Doppler ultrasound data. *Medical Engineering and Physics*, *19*(4), 327–335. https://doi.org/10.1016/S1350-4533(97)84634-6

Pennati., G., & Fumero., R. (2000). Scaling approach to study the changes through the gestation of human fetal cardiac and circulatory behaviors. *Annals of Biomedical Engineering*, *28*(4), 442–452.

Versmold, H. T., Kitterman, J. A., Phibbs, R. H., Gregory, G. A., & Tooley, W. H. (1981). Aortic blood pressure during the first 12 hours of life in infants with birth weight 610 to 4,220 grams. *Pediatrics*, *67*(5), 607–613.
